# Impact of Gaussian feathering on diagnostic metrics in tile-based micro-CT sinogram infilling

**DOI:** 10.1038/s41598-026-57319-2

**Published:** 2026-06-13

**Authors:** Falk L. Wiegmann, Nancy L. Ford

**Affiliations:** 1 Department of Physics and Astronomy, The University of British Columbia, Vancouver, BC Canada; 2 Department of Oral Biological and Medical Sciences, The University of British Columbia, Vancouver, BC Canada

**Keywords:** Engineering, Mathematics and computing

## Abstract

Tile-based sinogram infilling methods for micro-CT require merging overlapping output tiles to reconstruct full images. Gaussian feathering, the standard blending approach, produces visually seamless results but its effect on diagnostic image quality metrics has not been characterized. This study quantifies how Gaussian feathering affects noise power spectrum (NPS), modulation transfer function (MTF), noise equivalent quanta (NEQ), structural similarity index (SSIM), and peak signal-to-noise ratio (PSNR) compared to nearest priority blending, also known as Voronoi partition blending. We mathematically derived the variance reduction mechanism in Gaussian blending and experimentally compared both methods using sinograms infilled by DeepFill v2 as a representative generative inpainting model, applied to a micro-CT quality assurance phantom reconstructed from 50% undersampled data. Gaussian feathering reduced variance by up to 19.6% at overlap centers, causing NPS reduction of up to 13.8% at low spatial frequencies and NEQ inflation of up to 38.5%. MTF showed mixed effects with improvements at low frequencies but reductions at high frequencies. SSIM and PSNR showed statistically significant differences: sinogram PSNR differed by 0.05 dB (*p = 0.003*) and reconstruction SSIM by 0.009 (*p = 0.036*), both with small effect sizes. In this experimental configuration, Gaussian feathering distorted diagnostic metrics through a variance reduction mechanism, while standard fidelity metrics detected only subtle changes. Nearest priority blending may be preferred when diagnostic metrics are used to validate infilling methods, pending broader empirical validation.

## Introduction

Generative deep learning models are increasingly applied to micro-computed tomography (micro-CT) sinogram infilling, where they reconstruct missing or corrupted data caused by metal artifacts, detector gaps, or sparse-view acquisition^1–3^. These models synthesize plausible image content that can improve diagnostic image quality when the underlying sinogram is incomplete.

However, these generative models typically operate on fixed-size input patches, often 256 *×* 256 or 512 *×* 512 pixels. When processing clinical sinograms that exceed these dimensions, a tiling approach becomes necessary^4^: the sinogram is divided into overlapping tiles, each tile is processed independently, and the results are merged in the sinogram domain prior to reconstruction. This tile-based workflow requires a blending strategy to combine overlapping regions.

The standard approach for tile merging is Gaussian feathering, a technique formalized in the 1980s for panoramic image stitching^5^. This method applies distance-weighted averaging in overlap regions, producing visually seamless transitions between adjacent tiles. The technique has been widely adopted in image processing pipelines due to its effectiveness at eliminating visible seams^6^.

In micro-CT imaging, objective, task-based evaluation frameworks such as the noise power spectrum (NPS), modulation transfer function (MTF), and noise equivalent quanta (NEQ) are critical for evaluating image quality and diagnostic accuracy^7,8^. NPS characterizes the spatial frequency distribution of noise texture, MTF quantifies spatial resolution, and NEQ combines both to assess overall image quality. In contrast, the deep learning literature typically evaluates infilling methods using the structural similarity index (SSIM) and peak signal-to-noise ratio (PSNR)^9^. SSIM captures perceptual similarity through local comparisons of luminance, contrast, and structure, while PSNR quantifies pixel-wise reconstruction error^9^.

We hypothesized that the weighted averaging inherent in Gaussian feathering would reduce image variance in overlap regions, thereby affecting noise-based metrics. This study investigates this hypothesis by comparing Gaussian feathering to nearest priority blending, a method that selects pixels from the nearest tile center without averaging by essentially stitching together the raw tiles, preserving original noise characteristics. This is conceptually similar to a Voronoi-based selection of patch content^10,11^.

Gaussian feathering itself is well established in image mosaicing; however, its effect on noise statistics and diagnostic micro-CT metrics has not previously been derived or quantified. The contribution of this work is methodological rather than algorithmic: we do not propose a new infilling model, but instead characterize how a routine post-processing step (tile blending) can systematically distort the metrics used to evaluate such models. Specifically, the purpose of this study is to: (1) mathematically derive the variance reduction mechanism in Gaussian blending, (2) quantify the impact on NPS, MTF, and NEQ using micro-CT sinograms infilled with DeepFill v2^12,13^ as a representative model, and (3) determine whether SSIM and PSNR are sensitive to these blending-induced changes. To our knowledge, this is the first study to analytically derive and experimentally characterize how tile blending can alter diagnostic metrics (NPS, MTF, NEQ) in micro-CT sinogram infilling, and to evaluate whether conventional fidelity metrics (SSIM, PSNR) are sensitive to these distortions.

## Results

### Variance reduction in overlap region

**Fig. 1:**
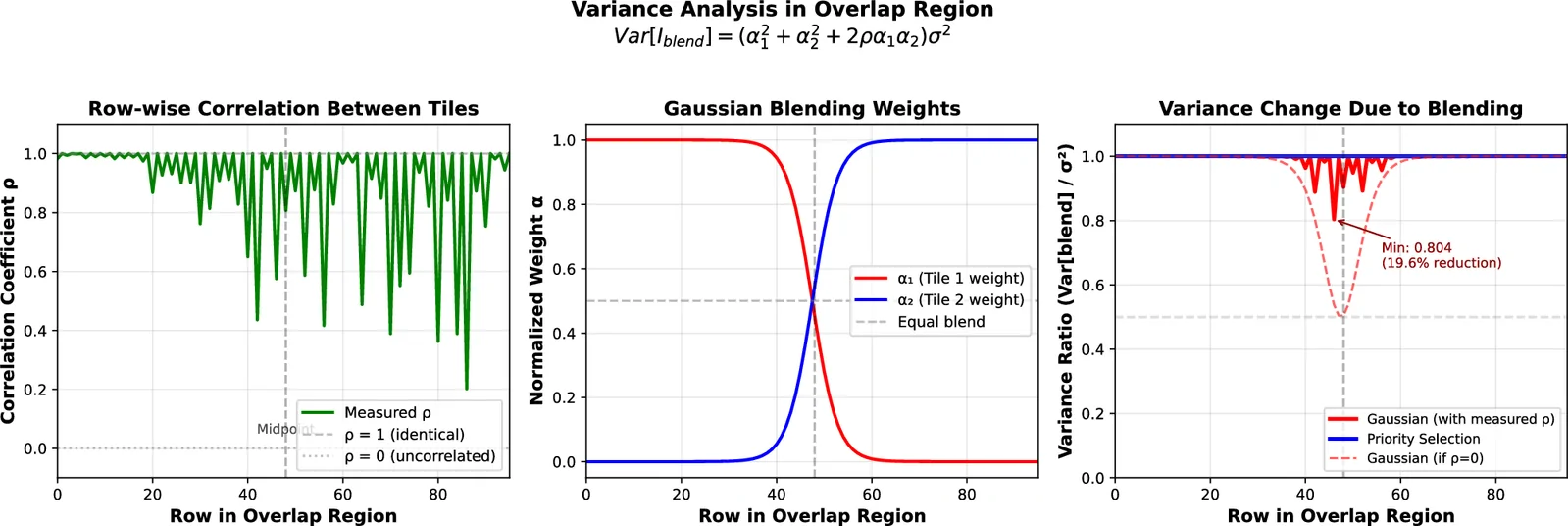
Variance analysis in overlap region for a single representative overlap region. (**a**) Row-wise correlation coefficient *ρ* between tiles. (**b**) Gaussian blending weights $\alpha _1$ and $\alpha _2$. (**c**) Variance ratio showing minimum of 0.804 (19.6% reduction) at overlap center for Gaussian blending, while Priority Selection maintains ratio of 1.0.

To characterize how Gaussian blending differs from nearest priority selection, we first examined variance behavior in the overlap region. Gaussian blending systematically reduced variance across the 96-pixel overlap, with the magnitude depending on inter-tile correlation. At the overlap center, where both tiles contribute equally ($\alpha _1 = \alpha _2 = 0.5$; Fig. 1b), the variance ratio $\text {Var}(I_{\text {blend}})/\sigma _{\text {pix}}^2$ reached a minimum of 0.804, representing a 19.6% reduction compared to nearest priority blending, which maintained constant variance throughout (Fig. 1c). The row-wise correlation coefficient *ρ* between tiles ranged from 0.2 to 1.0 across the dataset (Fig. 1a), modulating the degree of variance reduction according to Eq. (6). A correlation coefficient of 1 is observed every second row, corresponding to the 50% undersampling, meaning every second row is the ground truth.

### Frequency domain analysis

**Fig. 2:**
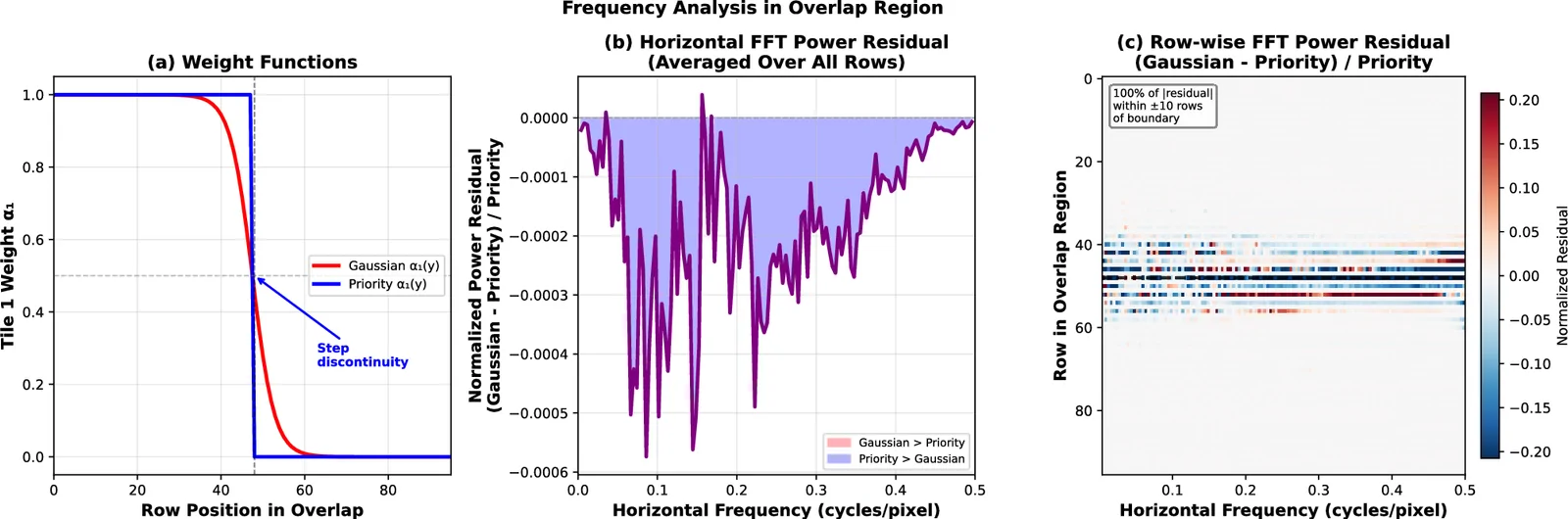
Frequency analysis in overlap region. (**a**) Weight functions comparing Gaussian smooth transition to Priority step discontinuity. (**b**) Horizontal FFT power residual (Gaussian − Priority) averaged over all rows, showing consistent power reduction. (**c**) Row-wise FFT power residual heatmap showing effect concentrated within ±10 rows of boundary.

Given that Gaussian blending reduces variance through weighted averaging, we next examined whether this effect manifests uniformly across spatial frequencies. The horizontal Fast-Fourier-Transform (FFT) power residual averaged across all rows (Fig. 2b) reveals that Gaussian blending consistently produces lower power than priority selection across all frequencies, with the largest reduction occurring between 0.05–0.15 cycles/pixel. Gaussian feathering does not act as a simple low-pass or high-pass filter; rather, it reduces power across a broad frequency range.

The spatial extent of this effect is confined to the blending region: the row-wise FFT power residual heatmap (Fig. 2c) shows that 100% of the power residual is concentrated within ±10 rows of the tile boundary. The weight functions (Fig. 2a) illustrate the underlying cause, contrasting the smooth Gaussian transition with the step discontinuity of priority selection.

### Effect of Gaussian *σ* parameter

**Fig. 3:**
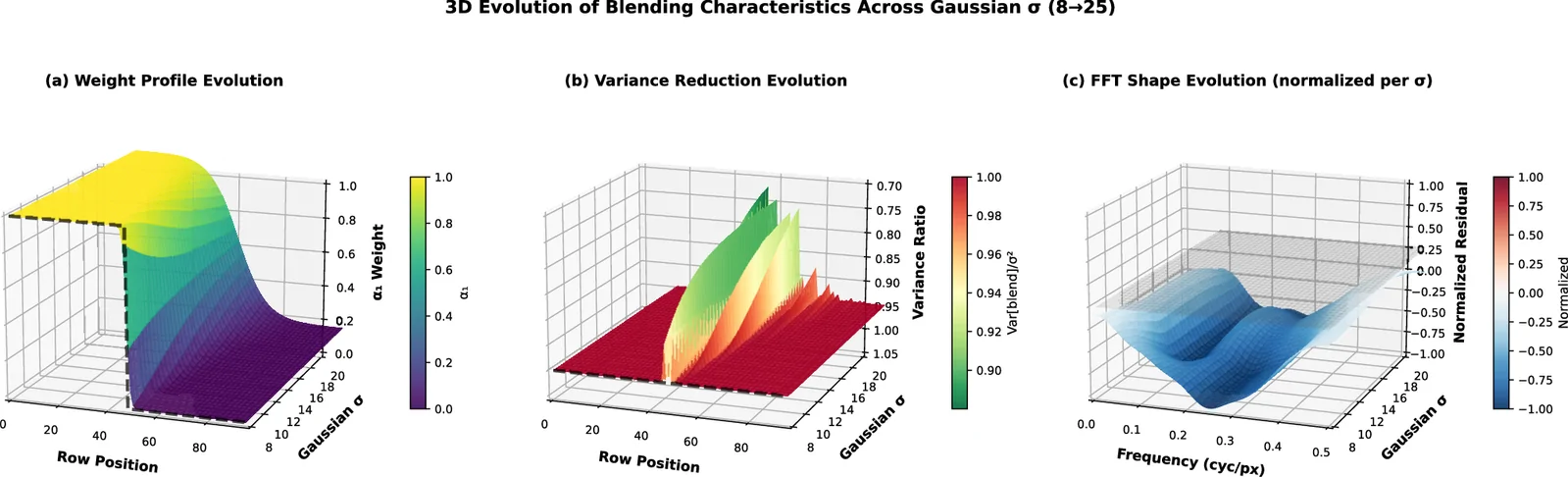
3D evolution of blending characteristics across Gaussian *σ* (8*→*25). (**a**) Weight profile evolution. (**b**) Variance reduction evolution showing increased reduction as *σ* increases. (**c**) FFT shape evolution (normalized per *σ*) showing consistent frequency residual distribution.

The Gaussian kernel width *σ* controls the sharpness of the blending transition, raising the question of how this parameter influences the observed variance and frequency effects. Decreasing *σ* produces sharper weight transitions that approach the binary selection of nearest priority blending (Fig. 3a), thereby diminishing the variance reduction effect (Fig. 3b). Conversely, increasing *σ* expands the region over which both tiles contribute and intensifies averaging near the overlap center, driving variance reduction toward its theoretical limit of 50% when *ρ = 0*.

Despite these magnitude changes, the frequency residual shape remains relatively consistent across *σ* values when normalized (Fig. 3c). This indicates that the spectral distribution of the smoothing effect is preserved regardless of blending sharpness. The Gaussian blending kernel does not approach a low-pass or high-pass filter at any *σ*.

### Impact on diagnostic metrics

**Fig. 4:**
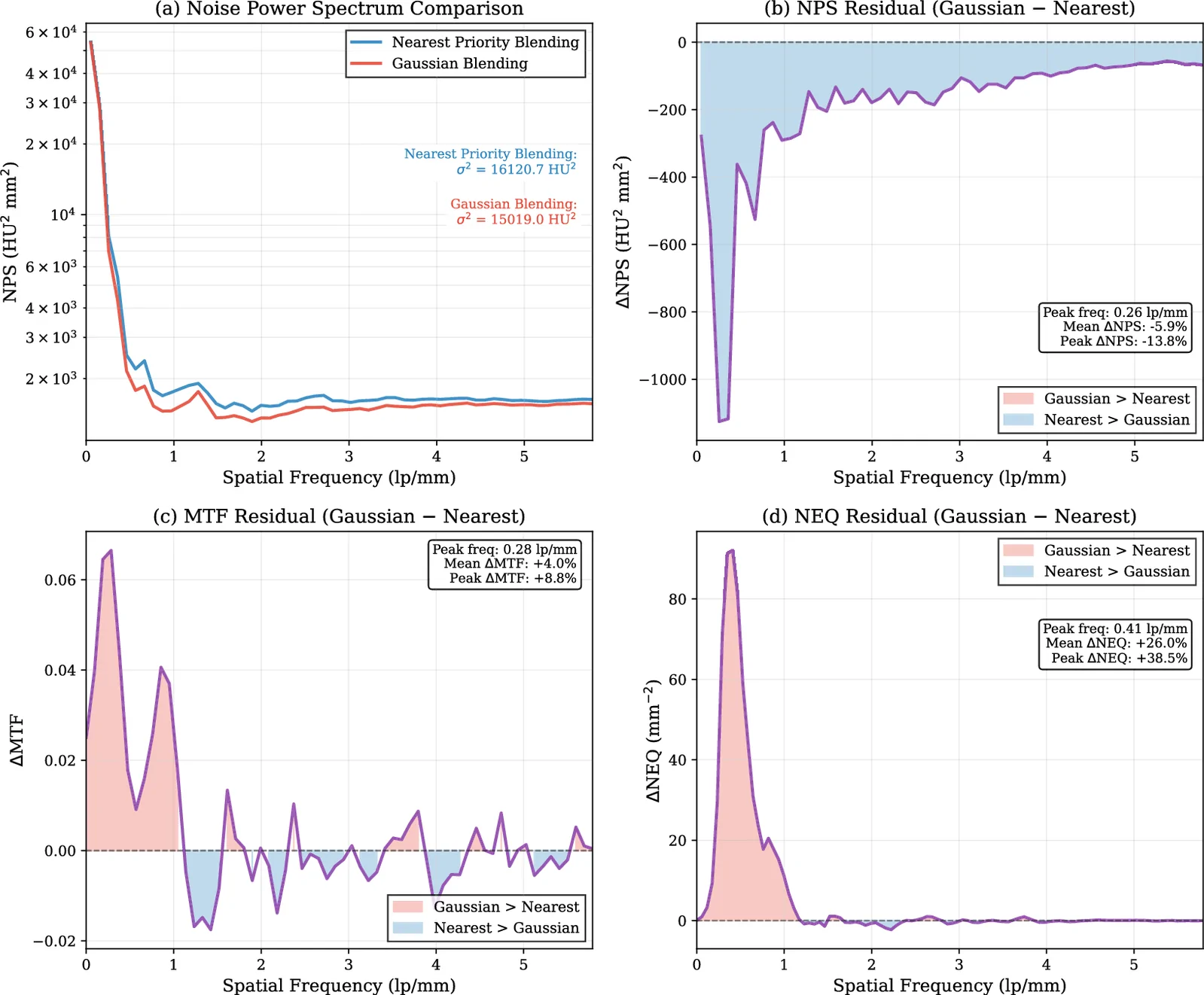
Diagnostic metrics comparison for DeepFill v2. (**a**) NPS curves showing Nearest Priority ($\sigma ^2 = 16121$ HU$^2$) vs Gaussian ($\sigma ^2 = 15019$ HU$^2$); (**b**) NPS residual with peak *Δ*NPS of *-13.8%* at 0.26 lp/mm. (**c**) MTF residual showing *+8.8%* peak at low frequencies; (**d**) NEQ residual with *+38.5%* peak inflation at 0.41 lp/mm.

The variance and frequency domain effects documented above have direct implications for diagnostic image quality metrics used to evaluate infilling methods. For NPS, Gaussian blending produced $\sigma ^2 = 15019$ HU$^2$ compared to $\sigma ^2 = 16121$ HU$^2$ for nearest priority, a mean reduction of 5.9%, with a peak reduction of 13.8% at 0.26 lp/mm (Fig. 4a,b). This effect was concentrated at low spatial frequencies (0–1 lp/mm), consistent with the broad-spectrum power reduction observed in the frequency domain analysis.

MTF showed mixed effects (Fig. 4c): mean *Δ*MTF was *+4.0%* with a peak of *+8.8%* at low frequencies, but MTF was reduced at high frequencies. This pattern suggests Gaussian blending may reduce visibility of small structures while enhancing low-frequency contrast.

The combined effect on NEQ was substantial (Fig. 4d): mean *Δ*NEQ was *+26.0%* with a peak of *+38.5%* at 0.41 lp/mm. Because NEQ *∝* MTF$^2$/NPS, the reduced NPS denominator inflates NEQ even when MTF changes are modest.

### SSIM and PSNR analysis

The variance reduction from Gaussian blending affects not only diagnostic metrics but also the fidelity metrics commonly reported in the deep learning literature. To quantify this effect, we compared SSIM and PSNR between Gaussian and nearest priority blending in both sinogram and reconstruction domains (Table 1). Statistical significance was assessed using *z*-tests, while practical significance was evaluated using Cohen’s *d*^14^. Tests were performed on the difference in the fidelity metrics between either blending method, with a null hypothesis that no change is detected between either blending method.

**Table 1:** SSIM and PSNR comparison between Gaussian and nearest priority blending.

Domain	Metric	Gaussian	Priority	*Δ*	*z*	*p*	Cohen’s *d*
Sinogram	SSIM	0.817	0.816	0.0009	1.22	0.22	0.04
Sinogram	PSNR (dB)	32.74	32.69	0.05	2.94	0.003$^*$	0.09
Reconstruction	SSIM	0.778	0.769	0.009	2.09	0.036$^*$	0.23
Reconstruction	PSNR (dB)	23.68	23.46	0.22	1.61	0.11	0.17

$$^*$$Statistically significant (*p < 0.05*).

Gaussian blending produced statistically significant improvements in sinogram PSNR (*p = 0.003*) and reconstruction SSIM (*p = 0.036*). However, the effect sizes are small: the same blending operation that produces a reconstruction SSIM difference of just 0.009 causes NPS reductions of up to 13.8% and NEQ inflation of up to 38.5%. SSIM and PSNR are therefore largely insensitive to the diagnostic metric distortions associated with Gaussian feathering in this configuration.

This matters because both blending methods process identical infilled content, so the same model output yields different metric values depending solely on how tiles are merged. The shift is large for the diagnostic metrics (up to 13.8% in NPS and 38.5% in NEQ) but too small in SSIM and PSNR to flag the underlying change, an issue compounded by inconsistent reporting of tiling and blending strategies in the medical physics literature^15^.

### Qualitative comparison of reconstructed images

**Fig. 5:**
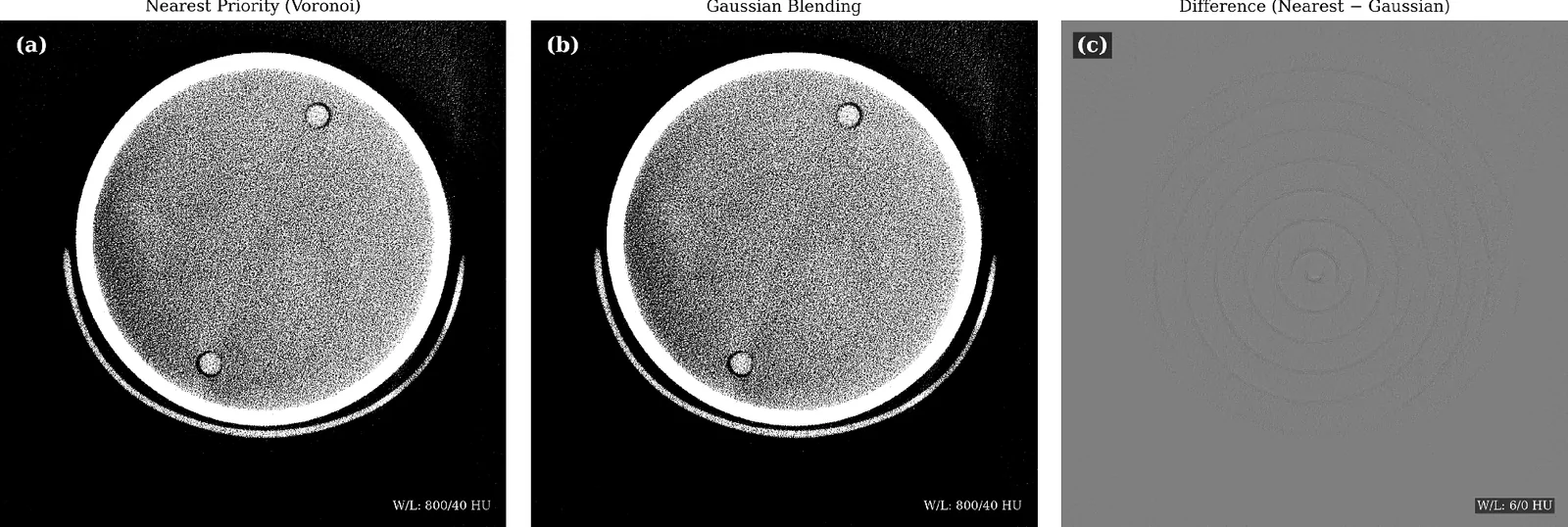
Qualitative comparison of reconstructed images (filter cutoff 0.334). (**a**) Nearest priority (Voronoi) blending (W/L = 800/40 HU). (**b**) Gaussian blending (W/L = 800/40 HU). (**c**) Difference image (Nearest − Gaussian, W/L = 6/0 HU) showing subtle structured ring patterns spaced at the tile stride.

To complement the quantitative analysis, Fig. 5 shows a representative reconstructed slice using both blending methods, reconstructed with a Ram-Lak filter cutoff of 0.334, matching the Nyquist limit of the reconstruction grid (detector pixel spacing/reconstruction voxel size *= 0.0284/0.085*). The two reconstructions are visually indistinguishable (Fig. 5a,b). The difference image (Fig. 5c) confirms that blending-induced differences are subtle: the maximum absolute difference is 1.0 HU with a mean of 0.07 HU (without the matched filter cutoff, the unfiltered blending effect produces a maximum of 3.9 HU and mean of 0.23 HU). The ring patterns in the difference image are spaced at an average of 66 reconstruction voxels, matching the predicted mapping of the horizontal sinogram tile stride (224 detector pixels, corresponding to a tile width of 256 minus 32-pixel overlap) to reconstruction space: $224 \times (0.0284\,\text {mm}/1.139) / 0.085\,\text {mm} = 65.7$ voxels, directly confirming the rings originate from tile boundary blending. A finer ring pattern from the generative model not capturing sinogram geometry is visible equally in both reconstructions and cancels in the difference: the relevant quantity is the inter-tile correlation *ρ* that the infilling introduces, not the geometric fidelity of the infilling itself. The spatially correlated nature of the blending differences explains why NPS captures the effect while SSIM and PSNR, which average over the full image, do not.

## Discussion

In this experimental configuration, Gaussian feathering introduced systematic distortion of diagnostic image quality metrics while producing visually seamless tile merging. Weighted averaging of overlapping pixel values reduces local variance, which propagates through to NPS and subsequently NEQ calculations.

The 13.8% peak NPS reduction and 38.5% peak NEQ inflation observed here are sizeable. Whether the choice of blending method would alter the relative ranking of specific infilling models in a practical comparative study has not been demonstrated here and requires a dedicated follow-up experiment with multiple models evaluated under both blending schemes. The practical impact of these distortions therefore depends on the models being compared: for two models with closely aligned fidelity (e.g., similar SSIM scores), the blending-induced distortion could be of the same order as the inter-model differences and could plausibly alter rankings, whereas for models that differ substantially in performance the relative ordering would be less affected. The present findings establish that such a comparison is warranted, but do not directly establish that conclusions about model performance would change in any specific scenario.

These distortions also have implications for task-based image quality assessment. Model observers such as the Channelized Hotelling Observer^16^, which use NPS and MTF as inputs to predict human observer performance in detection tasks, could yield inflated detectability indices when applied to Gaussian-blended images. This could lead to overoptimistic assessments of infilling method performance in clinically relevant detection tasks^17^.

These findings apply specifically to the evaluation of tile-based infilling methods. For purely visual applications where quantitative metrics are not critical, Gaussian feathering remains appropriate due to its superior perceptual continuity. However, when diagnostic metrics are used to validate or compare medical imaging algorithms, nearest priority blending may be a preferable choice to preserve noise characteristics. Extensions to nearest priority blending such as minimum error boundary cuts^18^, which find optimal seam paths through overlap regions, may further reduce visible discontinuities but introduce non-uniform sampling patterns whose effects on diagnostic metrics remain unexplored.

The scope of this work is an analytical derivation of the variance reduction mechanism, combined with an experimental characterization of its magnitude in one representative configuration: a single infilling model (DeepFill v2), a single tile geometry, a single undersampling pattern, and a single QA phantom. The findings reported here should therefore be interpreted as a demonstration of the mechanism rather than as a broad empirical survey of its magnitude across the space of possible configurations. Analytical generality does not replace experimental validation, and the magnitude of the effect under conditions other than those tested here remains an open empirical question for future work.

The magnitude of variance reduction depends primarily on the inter-tile correlation, which relates to model fidelity, with lower-fidelity models producing less correlated tiles and greater variance reduction. DeepFill v2^12,13^ (SSIM *≈* 0.8) is typical for current infilling models^1–3,19–22^, but this relationship warrants further investigation. The sensitivity to the Gaussian blending parameter *σ* is characterized in Fig. 3, where the variance reduction magnitude scales with *σ* while the spectral shape is preserved. The per-pixel mechanism (Eq. 6) is independent of tile size and overlap width. These parameters instead control the spatial extent of the distortion, with smaller tiles or larger overlaps amplifying the cumulative effect on NPS and NEQ.

Regarding generalizability beyond the QA phantom: NPS and MTF measurement require spatially uniform regions and known edge geometry, respectively^7^, conditions that cannot be reliably obtained from anatomical data. The variance reduction mechanism (Eq. 6) is a general analytical property of weighted averaging, predicted by the framework to occur whenever *ρ < 1*. The uniform geometry of the QA phantom yields one specific distribution of inter-tile correlations. For anatomically complex data, the inter-tile correlation could plausibly be lower (if increased task difficulty produces more variable model outputs across tiles) or higher (if structural constraints lead the model toward convergent outputs across tiles); the direction cannot be predicted without experimental validation. The framework predicts only that the variance reduction will scale with whatever *ρ* is observed under such conditions. Because the mechanism depends only on blending weights and inter-tile correlation, and not on the nature of the missing data, it is predicted to occur in any tile-based infilling pipeline, including those used for metal artifact correction, defective detector compensation, or reduced angular sampling. These predictions have not been empirically verified and are offered as hypotheses for future work.

Gaussian feathering reduced variance by up to 19.6% at overlap centers through weighted averaging of correlated tile content, propagating to up to 13.8% NPS reduction at low spatial frequencies and 38.5% NEQ inflation. These distortions could affect metric values in comparative evaluations of infilling methods, particularly between models of similar fidelity, although whether this would alter conclusions in any specific comparison requires a dedicated study. SSIM and PSNR showed statistically significant differences between blending methods with small effect sizes that do not reflect the magnitude of the underlying diagnostic distortions. This suggests that, at least in configurations comparable to the one studied here, relying solely on SSIM and PSNR may mask clinically significant changes in noise and resolution characteristics. In the configuration examined, tile blending, often treated as a minor implementation detail, can systematically distort diagnostic metrics used to evaluate infilling methods, while standard fidelity metrics show limited sensitivity to these distortions.

## Methods

### Micro-CT data acquisition and infilling

Sinogram data were acquired from an mCTP 610 quality assurance phantom (Shelley Automation Inc, Canada) using an eXplore CT 120 cone-beam micro-CT scanner (Trifoil Imaging, USA). Acquisition parameters were: 80 kVp tube voltage, 40 mA tube current, 410 projections over $360^{\circ }$ ($0.88^{\circ }$ angular spacing), with a detector size of 3500*×*2296 pixels and detector pixel spacing of 0.0284 mm. This yielded 2296 sinograms, each measuring 410 *×* 3500 pixels. The data were acquired by the authors at the University of British Columbia, Canada. As the mCTP 610 is a quality assurance phantom involving no human participants or animal subjects, no ethical approval or institutional permissions were required for data acquisition.

To generate controlled missing data for tile-based infilling, sinograms were retrospectively undersampled by 50%, removing every second projection row. This uniform angular undersampling provides a well-defined ground truth for metric computation and produces the type of incomplete sinogram data that tile-based generative methods are designed to process. The missing data were then infilled using the DeepFill v2 generative inpainting model^12,13^. DeepFill v2 employs gated convolutions and was applied using publicly available pre-trained weights without domain-specific fine-tuning, consistent with transfer learning practices common in medical imaging applications^23^. The model achieved reconstruction fidelity (SSIM *≈* 0.8) consistent with current sinogram infilling methods^1–3^, validating its use as a representative infilling model for this study.

### Tile configuration

The complete workflow proceeds as follows: sinograms are divided into overlapping tiles, each tile is independently infilled by the generative model, blending is applied in the sinogram domain to merge tiles, and finally the blended sinogram is reconstructed using the Feldkamp–Davis–Kress (FDK) algorithm^24^. Tiles measured 256 *×* 256 pixels with a vertical overlap of 96 pixels and vertical stride of 160 pixels, arranged in a 2 *×* 16 grid per sinogram. This configuration with a generous overlap is typical as it gives the infilling model more global context and sufficient overlap for blending the tiles^4^. Two adjacent tiles share a 96-pixel overlap region where blending occurs.

This resulted in approximately 73,500 tiles and 105,600 overlap regions analyzed using both blending methods in the sinogram and reconstruction domains.

**Fig. 6:**
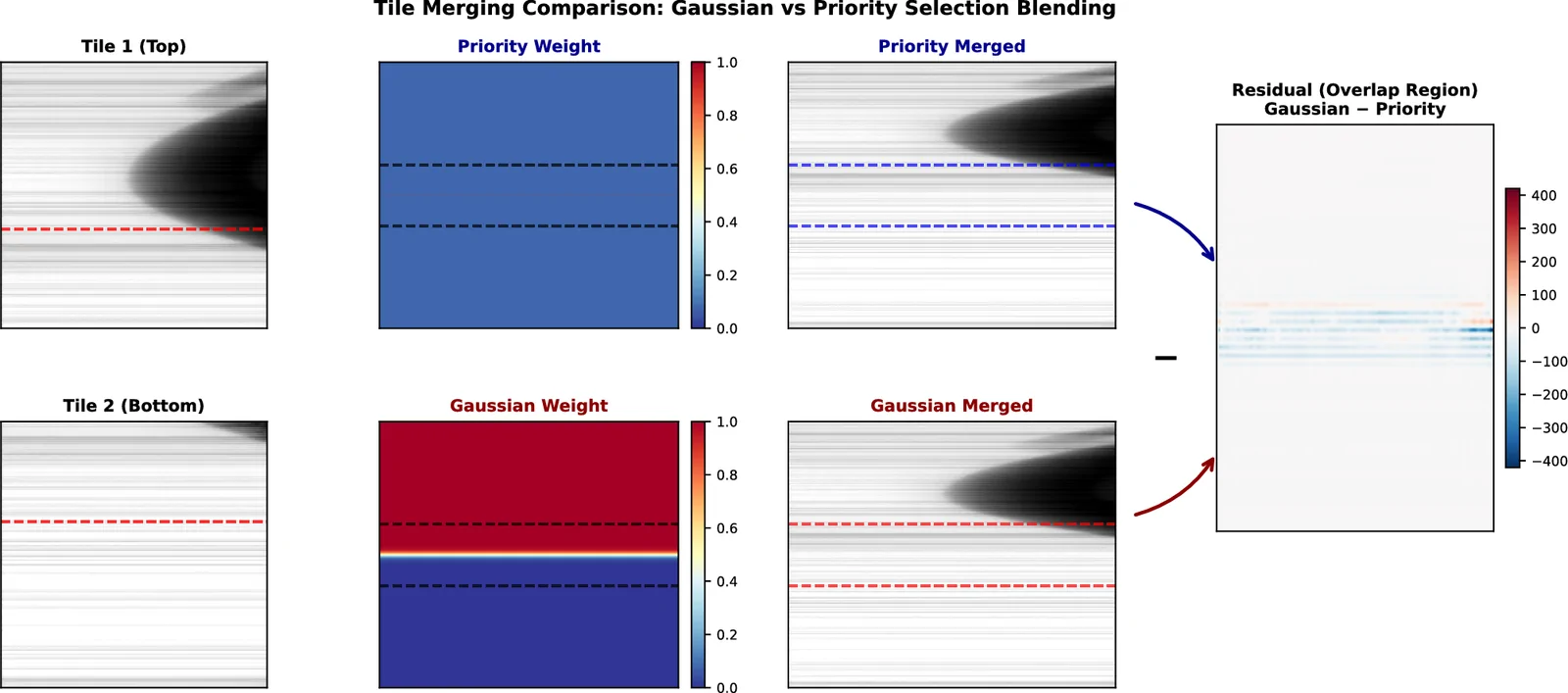
Tile merging comparison. Top row: Tile 1, priority weight map (binary selection), and priority-merged result. Bottom row: Tile 2, Gaussian weight map (smooth gradient), and Gaussian-merged result. The tile residual between methods (Gaussian − Priority) is shown on the right.

Figure 6 illustrates the tile merging process and the two blending methods compared in this study. The top row shows Tile 1, the priority weight map (binary selection), and the priority-merged result. The bottom row shows Tile 2, the Gaussian weight map (smooth gradient), and the Gaussian-merged result. The residual between the two approaches (Gaussian − Priority) is shown on the right, demonstrating the differences in how pixel values are combined within the overlap region.

### Gaussian feathering blending

In Gaussian feathering, the blended pixel intensity at position *p* in the overlap region is computed as a weighted average:

1 $$\begin{aligned} I_{\text {blend}}(p) = \frac{w_1(p) \cdot I_1(p) + w_2(p) \cdot I_2(p)}{w_1(p) + w_2(p)} \end{aligned}$$

where $I_1(p)$ and $I_2(p)$ are the pixel intensities from Tiles 1 and 2 respectively, and the weights are defined as:

2 $$\begin{aligned} w_i(p) = \exp \left( \frac{-d_i(p)^2}{2\sigma ^2}\right) \end{aligned}$$

Here $d_i(p)$ is the distance from position *p* to the edge of the overlap region for tile *i*, and *σ* is the Gaussian width parameter. We used *σ = 8*. Given our overlap width of 96 pixels, this choice ensures the weight decays to effectively zero at the tile boundaries (covering $\pm 6\sigma$), following standard practices for boundary smoothing in image mosaics^5^.

Defining normalized weights $\alpha _1(p) = \frac{w_1}{w_1 + w_2}$ and $\alpha _2(p) = \frac{w_2}{w_1 + w_2}$, the blended intensity becomes:

3 $$\begin{aligned} I_{\text {blend}}(p) = \alpha _1(p) \cdot I_1(p) + \alpha _2(p) \cdot I_2(p) \end{aligned}$$

### Nearest priority blending

As an alternative to Gaussian blending, nearest priority blending selects pixels from whichever tile has greater distance to its edge at that position:

4 $$\begin{aligned} I_{\text {priority}}(p) = {\left\{ \begin{array}{ll} I_1(p) & \text {if } d_1(p) > d_2(p) \\ I_2(p) & \text {otherwise} \end{array}\right. } \end{aligned}$$

This creates a binary selection at the overlap midpoint with no averaging, preserving original pixel values and noise characteristics. This approach is equivalent to a Voronoi partition where tile centers serve as seed points^10,11^.

### Variance analysis

Gaussian blending combines pixel values from two overlapping tiles through weighted averaging. This averaging has a predictable effect on noise: when two noisy signals are combined, their independent noise components average down—shrinking the expected variance— while correlated components add coherently. The degree of cancellation depends on how correlated the noise is between tiles.

For the blended signal $I_{\text {blend}} = \alpha _1 I_1 + \alpha _2 I_2$, assuming both tiles have equal variance $\sigma _{\text {pix}}^2$, the variance follows from standard properties of linear combinations of random variables (see Casella and Berger^25^, Section 4.5). To validate this equal variance assumption, we computed the noise variance ratio between tiles for each row after subtracting the known ground truth signal; when weighted by the blending weights, the mean ratio was *0.986 ± 0.030*, confirming effectively equal noise variance.

5 $$\begin{aligned} \text {Var}(I_{\text {blend}}) = \alpha _1^2\,\text {Var}(I_1) + \alpha _2^2\,\text {Var}(I_2) + 2\alpha _1\alpha _2\,\text {Cov}(I_1, I_2) \end{aligned}$$

Substituting $$\text {Var}(I_1) = \text {Var}(I_2) = \sigma _{\text {pix}}^2$$ and $$\text {Cov}(I_1, I_2) = \rho \sigma _{\text {pix}}^2$$:

6 $$\begin{aligned} \text {Var}(I_{\text {blend}}) = \left( \alpha _1^2 + \alpha _2^2 + 2\rho \alpha _1\alpha _2\right) \sigma _{\text {pix}}^2 \end{aligned}$$

where *ρ* is the correlation coefficient between corresponding pixels in the two tiles. This is a simplification possible due to the equal variance assumption. At the overlap center where $\alpha _1 = \alpha _2 = 0.5$, this simplifies to:

7 $$\begin{aligned} \text {Var}(I_{\text {blend}}) = \frac{1 + \rho }{2}\sigma _{\text {pix}}^2 \end{aligned}$$

Physically, for uncorrelated tiles (*ρ = 0*), variance is halved because independent noise fluctuations average down. For perfectly correlated tiles (*ρ = 1*), variance is preserved because identical fluctuations cannot cancel. In our infilled sinograms, the correlation ranged from 0.2 to 1.0, with *ρ = 1* occurring every second row where both tiles contain the original ground truth data from 50% undersampling.

### Diagnostic metrics

To quantify the impact of this variance reduction on diagnostic image quality, the following metrics were evaluated.

Noise power spectrum (NPS) was computed from reconstructed images as the Fourier transform of the noise autocorrelation function, characterizing noise texture across spatial frequencies^7,26^. Modulation transfer function (MTF) was measured using standard edge-spread function methods^27^. Noise equivalent quanta (NEQ) was calculated as MTF$^2$/NPS, representing the effective signal-to-noise ratio at each spatial frequency.

Structural similarity index (SSIM)^9^ and peak signal-to-noise ratio (PSNR) were computed between blended and ground truth images in both sinogram and reconstruction domains to assess overall image fidelity.

### Use of AI tools

Claude (Anthropic) was used to assist with manuscript preparation, including editing text and grammar review, and coding assistance during data analysis. All AI-generated content was reviewed, verified, and revised by the authors, who take full responsibility for the accuracy and integrity of the final manuscript.

## Data Availability

The micro-CT sinogram dataset analysed in this study is openly available in Zenodo (DOI: 10.5281/zenodo.18519787) under the folder Scan_1681. The code used to process the data and reproduce the analysis is publicly available on GitHub (UBC-Ford-lab/tile_blending_comparison_for_micro_ct_sinogram_infilling). The interim and derived results data generated during the study are available from the corresponding author on reasonable request.
